# From diet to hypothalamic dysfunction: Neuroanatomical and hormonal integration of the microbiota-hypothalamus-adipose tissue axis

**DOI:** 10.1007/s11154-026-10038-5

**Published:** 2026-04-21

**Authors:** Helena Dias de Freitas Queiroz Barros, Breno Picin Casagrande, Diana Dias Araújo, Thais Antonio Jose Mutran, Monica Marques Telles, Debora Estadella, Luciana Pellegrini Pisani

**Affiliations:** 1 Department of Biosciences, Federal University of São Paulo (UNIFESP), Institute of Health and Society (Campus Baixada Santista), 136 Silva Jardim St, Santos, 11015-020 SP Brazil; 2 Department of Physiology, Nutrition Physiology Division, Federal University of São Paulo (UNIFESP), Escola Paulista de Medicina (Campus São Paulo), 862 Botucatu St., 2nd floor, São Paulo, SP 04023-062 Brazil

**Keywords:** Obesity, Gut-brain axis, Gut microbiota, Hypothalamic inflammation, Short-chain fatty acids, Metabolic endotoxemia, Adipose tissue inflammation, Incretin therapy

## Abstract

Obesity is a chronic neurometabolic disorder characterized by low-grade systemic inflammation, adipose tissue dysfunction, and impaired central regulation of energy balance. This review summarizes mechanistic and translational evidence showing that diet-induced changes in the gut microbiota contribute to altered signaling along the gut-hypothalamus-adipose tissue axis. Obesogenic diets modify microbial metabolic activity by reducing short-chain fatty acid production, altering bile acid composition, and increasing endotoxin-related signaling, which together impair intestinal barrier function and promote metabolic endotoxemia. These signals reach the brain through endocrine (GLP-1, PYY), immune (cytokines, TLR4-dependent pathways), and neural (vagal/neuropod) routes, and are associated with hypothalamic microinflammation, impaired leptin and insulin signaling, and a persistent orexigenic drive. At the same time, adipose tissue undergoes hypertrophy and immunometabolic remodeling, reinforcing systemic inflammation and central dysregulation in a self-sustaining feedback loop. Incretin-based therapies, including GLP-1 and dual GIP/GLP-1 receptor agonists, act in part through modulation of this axis, including microbiota-related mechanisms. However, the frequent regain of weight after treatment discontinuation indicates that central and peripheral circuits are not fully reprogrammed. Viewing obesity as a disorder of disrupted inter-organ signaling highlights microbial metabolic pathways as relevant targets for more durable metabolic improvement.

## Introduction

Obesity is a chronic neurometabolic and multifactorial disease characterized by excessive fat accumulation, with impaired metabolic health, and compromised organ function [1]. Low-grade systemic inflammation is a defining hallmark of this condition, which is established locally and acts as a primary trigger for downstream metabolic and systemic secondary disruptions with widespread consequences [2, 3].

Obesity is associated with insulin and leptin resistance, dysregulated inflammatory responses, and altered glucose and lipid metabolism, increasing the risk of chronic non-communicable diseases (NCDs) [4, 5]. Recently, obesity has been reclassified, shifting focus beyond body weight. It was divided into preclinical, characterized by an increase in body adiposity that can be accompanied by alterations of cells, tissues, and organ structures, and minor manifestation; and clinical, characterized by impaired organ functions, clear clinical symptoms/complications, and limitations in basic daily activities [1].

The existence of a global obesity epidemic is common knowledge. According to the World Obesity Federation (2024), over 3.3 billion adults may be affected by 2035, compared with around 2.2 billion adults in 2020 [6]. This reflects a projected increase from 42% to more than 54% of the world’s population. Low-quality diet is also linked to NCDs and around 20% of deaths worldwide [7]. A cross-sectional study from Lu and colleagues (2025) has demonstrated a direct association between ultra-processed food consumption and increased risk of abdominal obesity, reinforcing the role of dietary processing and composition in adiposity related outcomes [8].

Although excessive caloric intake remains a basic component of obesity development, it does not fully explain the heterogeneity of metabolic outcomes observed across populations and individuals. Growing evidence indicates that obesity results from dysregulation of homeostatic and hedonic systems control of energy intake, expenditure, and nutrient partitioning [9]. These systems rely on continuous communication between central regulatory circuits and peripheral metabolic tissues.

The bidirectional interaction between the gastrointestinal tract, adipose tissue, and brain has emerged as a key regulatory axis. Rather than acting as isolated compartments, they form a network via which peripheral signals influence central energy regulation, and the central outputs model peripheral metabolic responses. Disruption of this communication is a hallmark of obesity and contributes to altered energy balance and metabolic and adipose tissue dysfunction [10, 11].

Beyond energy intake, dietary patterns shape how the body communicates within, influencing gut microbiota composition and function, immune responses, and metabolic regulation. Therefore, this manuscript aims to review the recent understanding of the gut microbiota-hypothalamus-adipose tissue axis in obesity pathophysiology, in which diet is proposed to act as the primary environmental driver.

## Diet, dysbiosis, and local metabolic reprogramming

Diet is a critical, and modifiable, determinant of gut microbiota structure and metabolic function. Both short-term dietary interventions and long-term habitual patterns can reshape microbial ecology, influencing not only taxonomic composition but, more importantly, microbial output and host signaling interfaces. The impact of diet extends beyond simplistic caloric excess. It includes macronutrient distribution and quality, that modulate microbial activity and host metabolic regulation. The obesogenic dietary patterns described over the years, and their effects on microbial metabolic pathways, are now reflected in the dietary index for gut microbiota (DI-GM) [12], which classifies patterns high in refined grains, red and processed meat, and > 40% energy intake from fat as unfavorable to gut health.

Those dietary patterns are closely linked to an obesogenic lifestyle, characterized by physical inactivity, insufficient sleep, high stress exposure, and environmental factors that favor overeating [13–15]. Obesogenic dietary patterns typically include high intake of refined sugars, salt, saturated animal fats, a high omega-6 to omega-3 ratio, refined grains, processed foods, and low consumption of fiber, fruits, vegetables, and nuts [7, 16]. Diet and lifestyle shape the composition of gut microbiota and contribute to the risk of increased inflammation and the development of metabolic syndrome, with diet quality playing a central role [17]. Recent population-based evidence further supports this relationship, demonstrating that dietary patterns associated with specific gut microbiota configurations are strongly linked to the prevalence of metabolic syndrome and systemic inflammatory markers [18].

Chandra et al. (2024) reports that obesity is more consistently characterized by microbial metabolic reprogramming than by taxonomic composition shifts [19]. These alterations include changes in SCFA production, bile acid transformation, branched-chain amino acid metabolism, and endotoxin-related signaling. Importantly, these functional outputs are more reproducible across cohorts than specific compositional signatures, reflecting a more stable association with host energy harvest, nutrient sensing, and metabolic phenotype [20–22]. 

This reprogramming is often driven by dietary deficiencies that have significant consequences for microbial metabolism, as the fermentation of non-digestible carbohydrates, fibers, by commensal bacteria is the primary source of SCFAs, such as acetate, propionate, and butyrate. Produced in the colon, they mediate critical host-microbiota interactions by regulating glucose and lipid metabolism, strengthening intestinal barrier integrity, and modulating the immune response [23, 24]. Alongside, the depletion of specific beneficial taxa, most notably *Akkermansia muciniphila*, serves as an indicative of metabolic dysfunction [25]. *A. muciniphila* acts as a critical regulator of host homeostasis, once its abundance inversely correlates with body weight and fasting blood glucose, and its proliferation is stimulated by IL-36, which plays a fundamental role in attenuating obesity and metabolic disorders [25, 26]. The protective influence of these taxa is tied to their roles in maintaining a healthy SCFA pool, either through direct production or by facilitating cross-feeding interactions that stabilize the metabolic output of the community [27, 28], hence their depletion compromises pathways essential for host homeostasis.

Among SCFAs, butyrate serves as the primary energy substrate for colonocytes and plays a critical role in maintaining epithelial integrity through tight junction stabilization and anti-inflammatory actions [29]. Propionate contributes to hepatic gluconeogenesis and stimulates secretion of anorexigenic gut hormones such as glucagon-like peptide-1 (GLP-1) and peptide YY (PYY) via activation of G-protein-coupled receptors GPR41 and GPR43 [30, 31]. Moreover, dysregulation in SCFAs metabolism, impaired production or signaling, has been associated with the opposite phenotypes, increased gut barrier permeability, metabolic endotoxemia, and associated diseases [32].

Beyond SCFAs, obesity‑associated dysbiosis impairs microbial conversion of primary bile acids into secondary ones [33], including lithocholic acid and deoxycholic acid, which are produced by specific gut bacteria, such as members of the Clostridium genus and *Parabacteroides distasonis*, many of which are often depleted in these cases [34]. Similarly, dysbiosis disrupts microbial tryptophan metabolism. High-fat diet-induced microbiota alterations reduce the production of the microbiota-dependent metabolite 5-hydroxyindole-3-acetic acid, reflecting impaired microbial conversion of tryptophan. This loss is associated with reduced microbial production capacity and impaired hepatic insulin signaling [35]. These metabolites act as ligands for host receptors such as TGR5 and the aryl hydrocarbon receptor (AHR), modulating inflammatory and metabolic signaling both peripherally and within the central nervous system [36–38]. Reduced TGR5 and AHR signaling has been implicated in hypothalamic dysfunction, where impaired bile acid and tryptophan-derived signaling may promote neuroinflammation and disrupt energy balance [37–39].

Under physiological conditions, the human gut microbiota is largely composed of the phyla Firmicutes and Bacteroidetes, which together account for the majority of bacterial cells, whereas Actinobacteria and Proteobacteria represent most of the remaining fraction [40, 41]. This taxonomic configuration was generally associated with metabolic homeostasis and intestinal barrier integrity. However, it should not be interpreted as a biomarker of health, since the relative abundances of these phyla show substantial inter-individual and inter-cohort variability and are poorly linked to specific phenotypes [42, 43].

Early reports of obesity-associated dysbiosis frequently emphasized an increased Firmicutes-to-Bacteroidetes (F/B) ratio as a defining feature. However, recent meta-analysis demonstrated a lack of reproducibility across cohorts, with no consistent pattern observed, despite the detectable changes in microbial diversity and composition [19]. A systematic review with meta-analysis further show high inter-study heterogeneity and inconsistent or non-significant differences at the phylum level, limiting its clinical value as a predictive marker [44]. In the same way, population-level data shows no association with obesity and BMI, also reporting lack of robustness from the F/B ratio [45]. In line with this, the field has been prioritizing functional metagenomics, while focusing on what the microbiota actually does, over taxonomic simplistic summaries [46, 47].

A similar limitation is observed with metrics of diversity. Alpha diversity tends to be reduced in obesity but with variable effect sizes and limited discriminatory value, while beta diversity consistently separates groups/profiles, indicating shifts in community composition despite overlapping within-sample diversity [47]. Consistently, pathway-level analyses outperform taxonomic metrics. A metagenomic functional index discriminated obesity more accurately than composition-based measures, although its performance decreased across independent cohorts, indicating population specificity [46].

In a multi-cohort analysis, individuals with obesity showed lower relative abundance of Firmicutes and higher Bacteroidetes compared to controls, opposite to earlier reports [47]. In the same study, obesity was associated with enrichment of species such as *Ruminococcus gnavus* and pathways related to glycosaminoglycan degradation, fructose and mannose metabolism, and lipopolysaccharide biosynthesis, whereas lean individuals showed higher abundance of *Akkermansia muciniphila* and pathways linked to carbohydrate transport and metabolism [47].

In T2DM, depletion of butyrate-producing taxa such as Faecalibacterium prausnitzii, Clostridium coccoides, and Eubacterium rectale, as well as the fiber-fermenting bacterium Ruminococcus faecis has been consistently reported, as reviewed by Hou and colleagues (2022), suggesting reduced microbial support for barrier and immune homeostasis, leading to a shift towards a pro-inflammatory cytokine profile [62]. Clinical evidence further supports the protective role of dietary fiber-driven microbial activity. In a randomized clinical trial, Chen and colleagues (2023) examined changes in gut microbiota composition and metabolic parameters in patients with T2DM following a high-fiber diet (17.9%, being 5.6% soluble and 12.3% insoluble). Post-intervention, significant increases were observed in general with SCFAs and lactic acid-producing bacteria, such as Lactobacillus, Akkermansia, Bifidobacterium, Bacteroides, Ruminococcus, and Blautia. Simultaneously, reductions were noted in opportunistic pathogens Erysipelatoclostridium, Megamonas, Prevotella, Klebsiella, and Desulfovibrio, which support that restoration of microbial fermentative capacity may mitigate obesity-associated metabolic disturbances [63].

Prolonged consumption of obesogenic diets promotes an increased abundance of gram-negative bacteria and elevated levels of lipopolysaccharides (LPS) in the gut, which was observed in a foundational study in mice [48] and subsequently confirmed in humans [49]. Sustained obesogenic feeding not only alters microbial diversity in the gut, but also reduces total bacterial load, contributing to gut barrier disruption and the establishment of a dysbiotic state [50, 51]. LPS through both direct epithelial actions and cytokine-mediated mechanisms, disrupt intestinal barrier integrity by altering the expression and cellular localization of tight junction proteins, including occludin, claudins, and zonula occludens-1 (ZO-1), thereby facilitating its translocation into the systemic circulation, as reviewed by Suzuki (2013).

Aside from fibers, dietary fat composition has profound effects on microbial ecology. Among dietary lipids, saturated fatty acids (SFAs) have been consistently associated with increased inflammatory responses and metabolic dysfunction, besides its lipotoxic effects [52]. In contrast, unsaturated fatty acids are generally linked to more favorable metabolic profiles [53, 54]. These distinct effects are partly mediated through interactions between dietary fats, gut microbiota composition, and innate immune signaling pathways. Early hypotheses proposed that long-chain saturated fatty acids (lcSFA) directly bind to Toll-like receptor 4/Myeloid differentiation factor 2/Cluster of differentiation 14 (TLR4-MD2-CD14) complex similarly to LPS. However, Lancaster and colleagues (2018) have shown that lcSFAs do not directly activate TLR4 but instead amplify its signal through a mechanism that requires prior priming (sensitization), typically by LPS [55]. Still, SFA causes an increase in inflammatory processes, even though it does not directly bind to TLR4 [56]. Li et al. (2020) propose that they mechanically promote the translocation of TLR4 into lipid rafts, which facilitates receptor dimerization within the TLR4/MD2 complex and subsequent endocytosis, promoting the inflammatory response.

Experimental studies further demonstrate that dietary fat composition shapes gut microbial configurations with distinct metabolic consequences. Caesar and colleagues (2015) showed how lard-based diets promote microbial configurations enriched in gram-negative taxa, including members of the *Desulfovibrionaceae* family within *Proteobacteria*, while reducing taxa commonly associated with metabolic homeostasis, such as *Lactobacillus*, leading to enhanced MyD88/TRIF-dependent TLR signaling and adipose tissue inflammation, indicating a microbiota-driven mechanism [57].

Consistently, diets enriched in SFAs, including lard and coconut oil, reduced overall microbial diversity and are associated with decreased *Bifidobacterium* and *Lactobacillus*, with concurrent increases in endotoxin-producing bacteria. These alterations coincide with insulin resistance and hepatic steatosis, whereas unsaturated oils induce more favorable microbial and metabolic profiles [58–60]. An integrative translational mouse and human analyses demonstrate that dietary fatty acid composition interacts with gut microbiota to influence hepatic lipid accumulation, with SFA-enriched diets associated with reduced abundance of *Akkermansia muciniphila* and enrichment of pro-inflammatory microbial signatures linked to steatosis and systemic inflammation [61].

Ultimately, gut microbiota composition exhibits substantial inter-individual variability influenced by long-term dietary patterns, cultural and environmental factors, and personal characteristics [64, 65]. Long-term dietary intake is a major determinant of community structure, with habitual macronutrient patterns associated with distinct enterotype-like configurations [66]. Although consistent taxonomic and functional trends are reported across metabolic and inflammatory diseases, the magnitude and specific microbial signatures associated with these conditions vary between individuals and populations [17, 67]. This heterogeneity emphasizes the need to interpret microbiota-disease associations within dietary and lifestyle contexts rather than as uniform microbial profiles.

## Transmission of microbial-derived signals beyond the gut

These diet-driven changes in microbial metabolic output are not confined to the intestinal lumen but are transmitted systemically through multiple signaling pathways, influencing host physiology [21, 68]. The impact of these signals depends not only on their molecular identity but also on the pathways through which they are transmitted. These signals are transmitted through three partially overlapping pathways: endocrine, immune-inflammatory, and neural pathways [69].

**Fig. 1:**
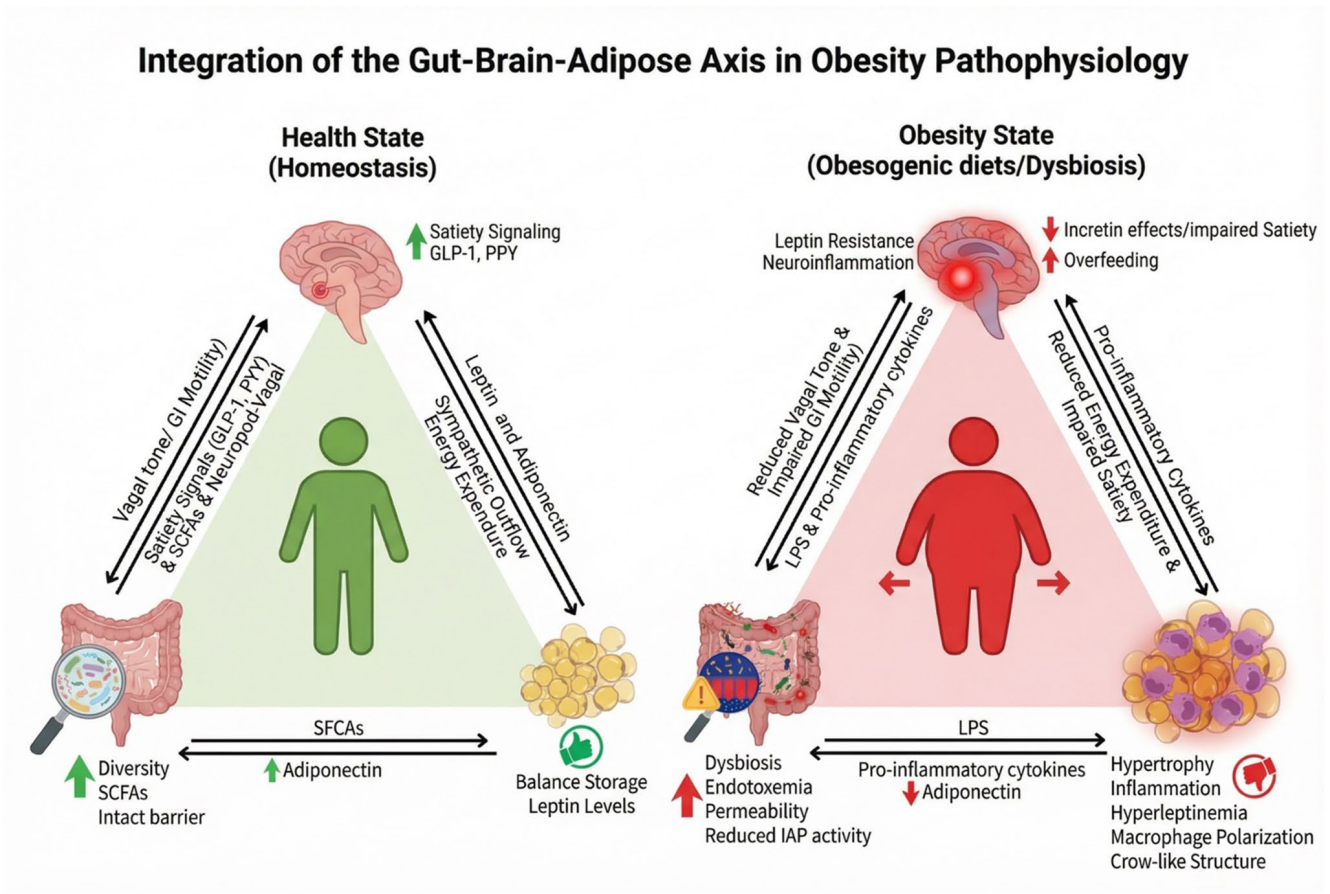
Physiological integration of gut-brain-white adipose tissue in health and obesity. The figure accurately illustrates the gut-brain-adipose tissue axis in both healthy (homeostasis) and obese states. Homeostasis: Balanced intestinal microbiota preserves gut barrier integrity and promotes short-chain fatty acid (SCFA) production. Circulating SCFAs support balanced lipid storage, restore leptin levels, and elevate adiponectin in white adipose tissue. Adipokines such as leptin and adiponectin reach the brain, establishing satiety signals through incretin action (PYY, GLP-1), which are also directly stimulated by intestinal SCFAs. Obese State - Obesogenic Diets/Dysbiosis: Dysbiosis compromises intestinal barrier integrity, increasing endotoxemia (circulating LPS). LPS reaches white adipose tissue, triggering low-grade chronic inflammation with macrophage polarization (M1), hyperleptinemia with leptin resistance, reduced adiponectin levels and activity, pro-inflammatory cytokine production, and adipocyte hypertrophy. These factors reach the hypothalamus, inhibiting incretin effects (PYY, GLP-1) and promoting overeating

Functionally, gut-brain communication comprises a rapid neural pathway, in which enteroendocrine neuropods synapse onto vagal afferents with millisecond precision, and a slower systemic pathway involving microbial products such as LPS and cytokine release that shape longer-term inflammatory responses [70, 71]. Sustained exposure to circulating cytokines and endotoxin can alter blood-brain barrier (BBB) permeability through endothelial and neuroimmune activation, including microglial inflammasome signaling and immune cell recruitment, thereby modulating central physiology over time [72, 73], as illustrated in Fig. 1.

Notably, the brain plays a significant role in gut function, regulating motility, the secretion of acid, bicarbonate, and mucus, and regulating intestinal fluids dynamics, which are essential for maintaining the mucus layer that supports commensal bacteria. Consequently, disruption of this axis can lead to alterations in the intestinal mucosa and microbiota composition.

Endocrine signaling represents a primary route through which gut-derived signals influence distant tissues. During food consumption, enteroendocrine cells (EECs) detect luminal nutrients and secrete key hormones, including peptide YY (PYY), cholecystokinin (CCK), glucagon-like peptide-1 (GLP-1), ghrelin, and leptin, which act on specific receptors in the hypothalamus and brainstem to modulate satiety, gastrointestinal motility, and insulin secretion [71, 74]. Growing evidence indicates that the microbiota can influence host behavior and appetite, including eating patterns and preferences, by modulating these satiety pathways [75, 76]. Microbial configurations can shift host preference for protein-to-carbohydrate ratios by modulating the synthesis of essential amino acids and the availability of plasma tryptophan, the critical precursor for central serotonin synthesis, which in turn regulates macronutrient intake [75].

This behavioral modulation complements the regulation of neuroendocrine signals; for instance, while certain bacterial species stimulate orexigenic ghrelin production, others, such as members of the genera *Bifidobacterium* and *Lactobacillus*, can inhibit its secretion [77]. Furthermore, in mice, *Akkermansia muciniphila* secretes a protein that binds to ICAM-1 on L-cells to induce GLP-1 secretion. This signaling also enhances systemic energy expenditure by upregulating uncoupling protein 1 (UCP1) in brown fat, thereby increasing thermogenesis [78].

Immune-inflammatory signaling provides a critical pathway linking intestinal activity to systemic metabolic regulation. Alterations in intestinal barrier integrity facilitate the detection of microbe-associated molecular patterns (MAMPs) by pattern recognition receptors (PRRs). As first demonstrated in the landmark studies by Cani et al. (2007, 2008), high-fat feeding promotes a 2 to 3-fold increase in circulating LPS, a state coined metabolic endotoxemia, which serves as a primary trigger for low-grade inflammation, weight gain, and insulin resistance.

Mechanistically, this process is initiated by a rapid decrease in the expression and activity of intestinal alkaline phosphatase (IAP) [79]. Specifically, high-fat diet consumption has been shown to induce a significant decrease in the expression and activity of IAP, a brush-border enzyme essential for dephosphorylating and neutralizing the lipid A moiety of LPS. Its deficiency directly impairs the gut’s ability to detoxify luminal endotoxins, promoting their translocation into the portal circulation [79–81].

Recent prospective data have further established that IAP deficiency is an independent risk factor for the development of metabolic syndrome and type 2 diabetes in humans, irrespective of obesity status [82]. Upon entering systemic circulation, gut-derived LPS activates the TLR4-MyD88 signaling axis and the NLRP3 inflammasome in hematopoietic cells, triggering the NF-κB and JNK cascades [48, 83]. These cascades result in the chronic release of pro-inflammatory cytokines, such as TNF-α, IL-6, and IL-1β, which can alter BBB integrity via microglial activation and peripheral inflammation–induced barrier permeabilization [72, 73]. This immune-mediated communication translates local gut disturbances into a systemic inflammatory state, influencing metabolic organs even in the absence of massive microbial translocation.

Neural pathways enable rapid transmission of gut-derived information to the central nervous system. EECs form direct synapse-like contacts with vagal afferent fibers, allowing luminal and mucosal signals to be relayed to the brainstem within seconds to minutes. This bidirectional communication is mediated in part by the enteric nervous system and vagal afferents, which respond to intestinal hormones, peptides, and microbial metabolites. Neural signals reach the brain, integrating gut-derived inputs to maintain metabolic homeostasis [84–86].

Among microbial metabolites, SCFAs stand out as key mediators. Beyond serving as energy for colonocytes, SCFAs modulate endocrine signaling and neurotransmitters such as serotonin (5-HT) and GABA [87, 88]. At the molecular level, SCFAs modulate gene expression through the inhibition of histone deacetylases (HDACs) [89, 90]. Histone acetylation by histone acetyltransferases (HATs) opens chromatin for transcriptional activity, while removal by HDACs leads to gene silencing [91]. Butyrate acts as an HDAC inhibitor, promoting histone hyperacetylation and the activation of specific gene clusters. In the brain, this mechanism upregulates neurotrophic factors such as brain- and glial-derived neurotrophic factors (BDNF and GDNF), facilitating DNA repair and exerting neuroprotective effects [92, 93]. In the gut, butyrate-driven HDAC inhibition increases the expression of tight junction genes, such as *TJP1* (ZO-1), *OCLN* (Occludin), and *CLDN* (Claudins), alongside the mucin gene *MUC2*, which are critical for maintaining barrier integrity and preventing the translocation of endotoxins [90, 94].

**Fig. 2:**
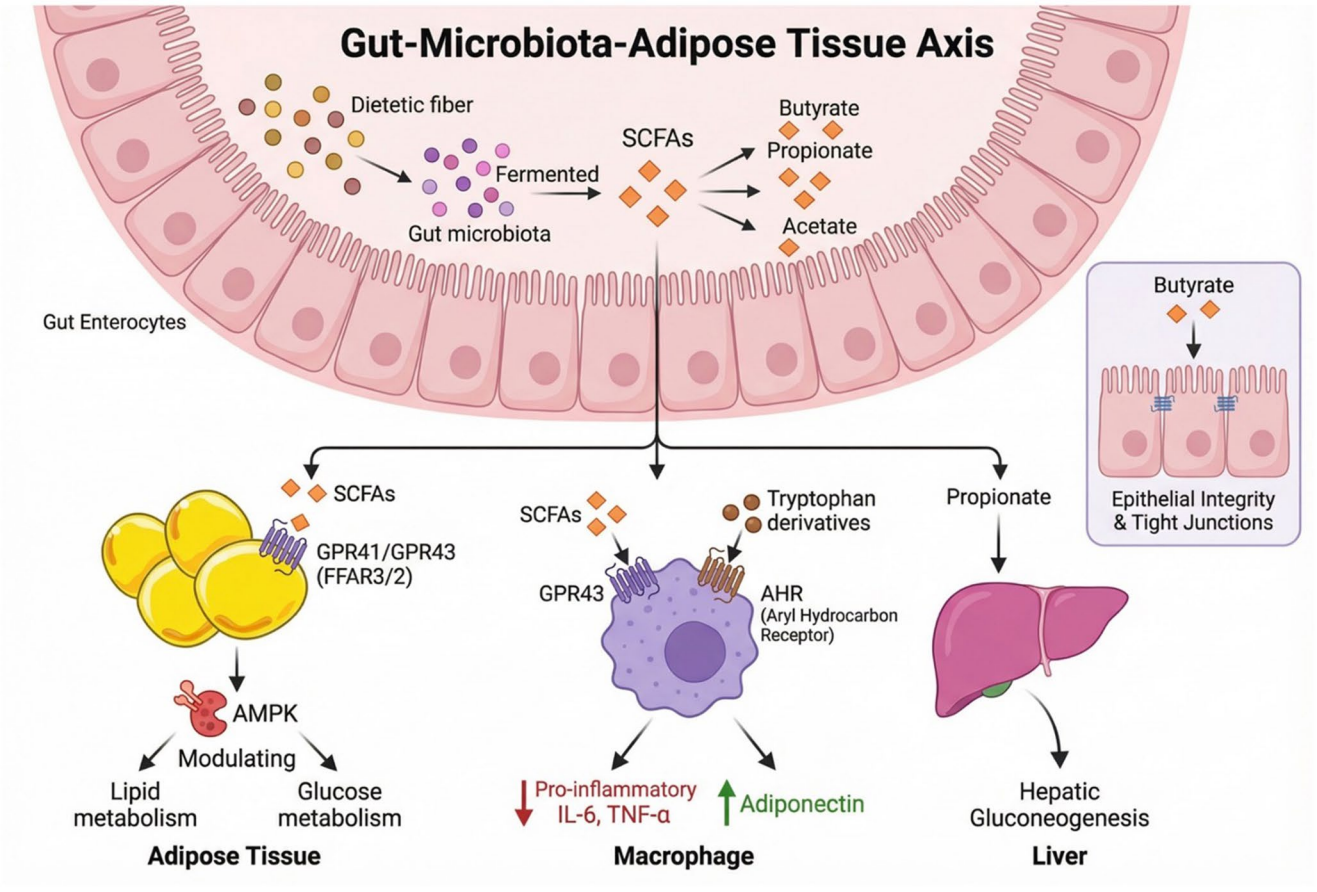
Gut microbiota-derived short-chain fatty acids in adipose tissue and immune regulation. This diagram depicts the production of short-chain fatty acids (SCFAs)—acetate, propionate, and butyrate, through microbial fermentation of dietary fiber in the intestine and their systemic metabolic effects. SCFAs are absorbed by intestinal epithelial cells and interact with G-protein-coupled receptors (GPR41/FFAR3, GPR43/FFAR2) in peripheral tissues, including adipocytes and immune cells. In adipose tissue, SCFA signaling modulates intracellular metabolic pathways, including AMP-activated protein kinase (AMPK), influencing lipid handling and glucose metabolism. In macrophages, SCFAs suppress pro-inflammatory cytokine production (e.g., IL-6) and promote anti-inflammatory responses via AhR/IL-10. In the liver, they inhibit gluconeogenesis. Together, these mechanisms illustrate how gut microbial metabolites act as signaling intermediates connecting the microbiota to host energy homeostasis

Furthermore, GPR43 and GPR41 are expressed in metabolically active tissues and immune cells, linking the microbiota to the gut-adipose tissue axis. Upon activation, these receptors modulate lipid and glucose metabolism, partly via regulation of AMPK and hormone-sensitive lipase (HSL) activity [95, 96], as shown in Fig. 2. This bidirectional axis is driven by microbial SCFAs, notably butyrate and propionate, activating PPAR-γ to regulate adipocyte differentiation lipid storage, and inflammatory status. Within this crosstalk, the microbiota regulates systemic leptin sensitivity via GLP-1 receptor signaling and maintains the balance between pro- and anti-obesity adipokines, while host adipokines such as leptin and adiponectin reciprocally shape the microbial composition [97, 98].

Microbial metabolites generated in the gut can enter systemic circulation and exert distal metabolic effects. Early evidence indicated that the intestinal microbiota enhances intestinal glucose absorption, leading to increased circulating glucose and insulin levels. These metabolic changes were shown to activate the transcription factors carbohydrate response element-binding protein (ChREBP) and sterol regulatory element-binding protein-1 (SREBP-1), thereby promoting lipogenic gene expression [99]. More recently, Berngentall et al. (2025) have refined this model by demonstrating that microbiota-diet interactions are required for carbohydrate-driven activation of hepatic lipogenic programs, including SREBP-1c–dependent de novo lipogenesis, highlighting the gut microbiota as a critical modulator of glucose-lipid metabolic coupling [100].

Vagal afferent signaling constitutes a major route through which microbial-derived signals from the gut are conveyed to central circuits. Experimental evidence demonstrates that gut microbes can influence brain function and behavior via the vagus nerve, and specialized circuits allow for the rapid transmission of luminal signals to the brainstem [101].

The microbiota influences the central nervous system through multiple parallel pathways, including vagal-independent neural routes and endocrine signals that modulate hypothalamic function in response to microbial metabolic activity. Propionate suppresses food intake and modulates systemic glucose homeostasis through a gut–portal–spinal circuit in which activation of FFAR3 receptors on periportal afferent nerves relays signals via the parabrachial nucleus to hypothalamic centers, including the arcuate nucleus, lateral hypothalamus, and paraventricular nucleus [102].

The microbiota also shapes endocrine pathways by triggering the release of liver-derived hormones through a gut microbiota-hepatic FGF21 axis. Recent evidence demonstrates that fermentable dietary polysaccharides modulate the gut microbiota, enriching beneficial taxa such as *Lactobacillus* and *Dubosiella*, to promote the production of SCFAs, particularly butyrate [103]. This microbial shift serves as an upstream trigger for the robust upregulation of hepatic FGF21, a pleiotropic hepatokine that coordinates energy expenditure, glucose-lipid homeostasis, and macronutrient preference [103–105]. FGF21 crosses the blood-brain barrier to act on its CNS co-receptor, β-Klotho, where it suppresses sweet and alcohol preference while increasing protein preference [106, 107].

Endocrine modulation also includes thyroid hormone (TH) signaling, which operates independently of direct neural transmission and is highly sensitive to nutritional status. Microbiota-derived metabolites influence iodothyronine deiodinase activity and thyroid hormone availability, modulating local triiodothyronine (T3) action within central metabolic circuits [108]. Microbiota-dependent regulation of bile acid composition further intersects with endocrine control. By modulating levels of tauro-β-muricholic acid, an FXR antagonist, the microbiota promotes intestinal FGF15 signaling and suppresses hepatic bile acid synthesis [109], linking microbial metabolism to systemic energy regulation and thyroid-related pathways. Consistently, a Mendelian randomization study supports an association between microbial composition and thyroid function, with specific taxa linked to altered thyroid status and susceptibility to dysfunction in humans [110].

In the ventromedial hypothalamus, T3 signaling in SF1 neurons coordinates peripheral metabolism through a dichotomic autonomic mechanism, simultaneously promoting hepatic *de novo* lipogenesis via parasympathetic outputs and brown adipose tissue thermogenesis via sympathetic activation [111, 112]. These central actions occur independently of systemic thyroid levels and position hypothalamic T3 signaling as a key effector through which microbial metabolic cues can influence whole-body energy balance.

Together, these observations indicate that the transmission of microbial signals occurs through an integrated network of neural, endocrine, and immune pathways. By incorporating vagal-independent spinal circuits, central integrative nodes such as orexin signaling, and endocrine modulators including FGF21 and T3, the gut microbiota emerges not as a passive contributor to nutrient processing, but as an active integrator of dietary inputs with host glucose and lipid metabolism, shaping hypothalamic function through diverse, predominantly non-inflammatory mechanisms that sustain metabolic and behavioral homeostasis.

## Immune-inflammatory signaling and hypothalamic vulnerability

Among the target organs of these signaling pathways, the hypothalamus is particularly sensitive to peripheral metabolic and inflammatory cues. The hypothalamus functions as the primary integrative center for the homeostatic regulation of food intake and energy balance. It continuously detects and integrates metabolic, hormonal, and neural signals originating from peripheral organs – including the gastrointestinal tract and adipose tissue – and translates this information into coordinated neuroendocrine and autonomic responses that govern feeding behavior [113, 114]. Within this region, the ARC is particularly susceptible to peripheral fluctuations due to its anatomical proximity to the median eminence, a circumventricular organ where the BBB is significantly more permeable [115].

Circadian rhythms in the gut microbiota and host metabolism, coordinated in part by CLOCK/BMAL1-dependent oscillations, interact with bile acid–FXR signaling to influence hypothalamic function. These temporal dynamics are disrupted by obesogenic diets, with downstream effects on nocturnal leptin sensitivity and morning brown adipose tissue thermogenesis. This topic has been reviewed in detail by Zhang and colleagues [116].

While metabolic endotoxemia, as established in Sect. 3, is often cited as the primary driver of hypothalamic dysregulation and LPS-induced neuroinflammation, recent evidence suggests a context-dependent reality. Chen and Gautron (2025) argue that the contribution of metabolic endotoxemia is highly nuanced, as physiological barriers and clearance mechanisms limit sustained systemic LPS exposure [117]. A quantitative study on BBB transport supports this view, showing that only around 0.025% of intravenous LPS enters the brain parenchyma, with most LPS remaining bound to the luminal surface of brain endothelial cells [118]. Notably, this lack of central penetration persisted even when a high intravenous dose of LPS (100 µg per mouse, 3–4 mg/kg) was administered. When administered intraperitoneally, this range has been shown to induce robust and long-lasting systemic inflammation and microglial activation with behavioral impacts [119].

Accordingly, activation of TLR4/CD14 in specific sites does not likely depend solely on chronic elevations of circulating LPS, but may instead arise from chronic localized intestinal sensing, transient postprandial dynamics, or alternative immune and neural signaling routes. Gut-derived inflammatory mediators (TNF-α, IL-6) weaken the BBB and activate endothelial cells, triggering local inflammatory signaling that propagates across the barrier without requiring major LPS translocation [120, 121]. Furthermore, EECs have been reported to express TLR4 that can sense luminal or mucosa-associated LPS, eliciting secretory and immune-related signaling responses [122].

**Fig. 3:**
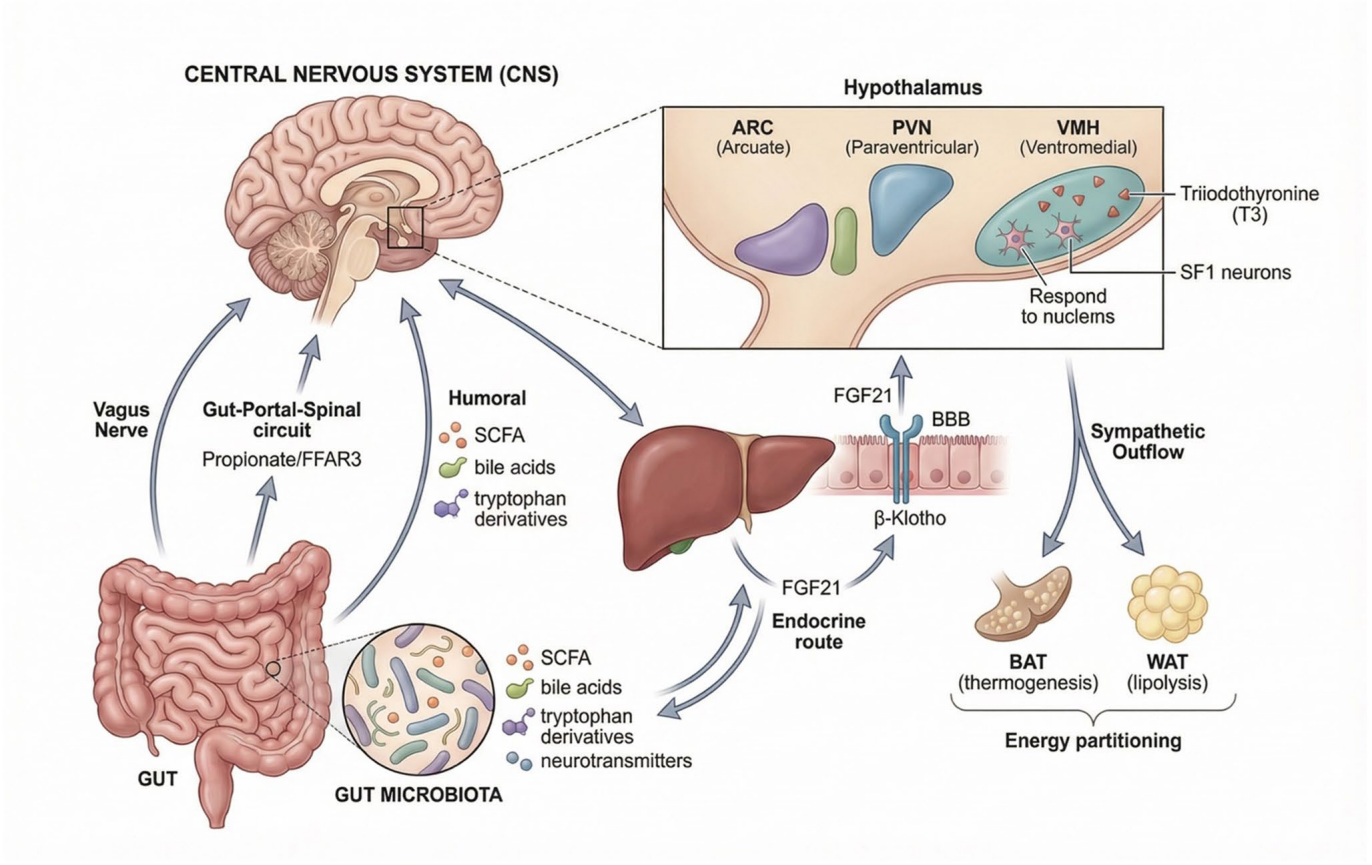
Multidirectional mechanisms of gut microbiota in the gut-hypothalamus-adipose tissue axis. The gut microbiota modulates hypothalamic function via parallel neural pathways (vagal and gut-portal-spinal), endocrine routes (hepatic FGF21, thyroid hormone signaling), and sympathetic outflow (PVH → BAT/WAT), integrating central control of thermogenesis and energy partitioning. ARC: arcuate nucleus; PVH: paraventricular nucleus; VMH: ventromedial hypothalamus; BAT: brown adipose tissue; WAT: white adipose tissue; FFAR3: free fatty acid receptor 3; β-Klotho: FGF21 co-receptor

**Fig. 4:**
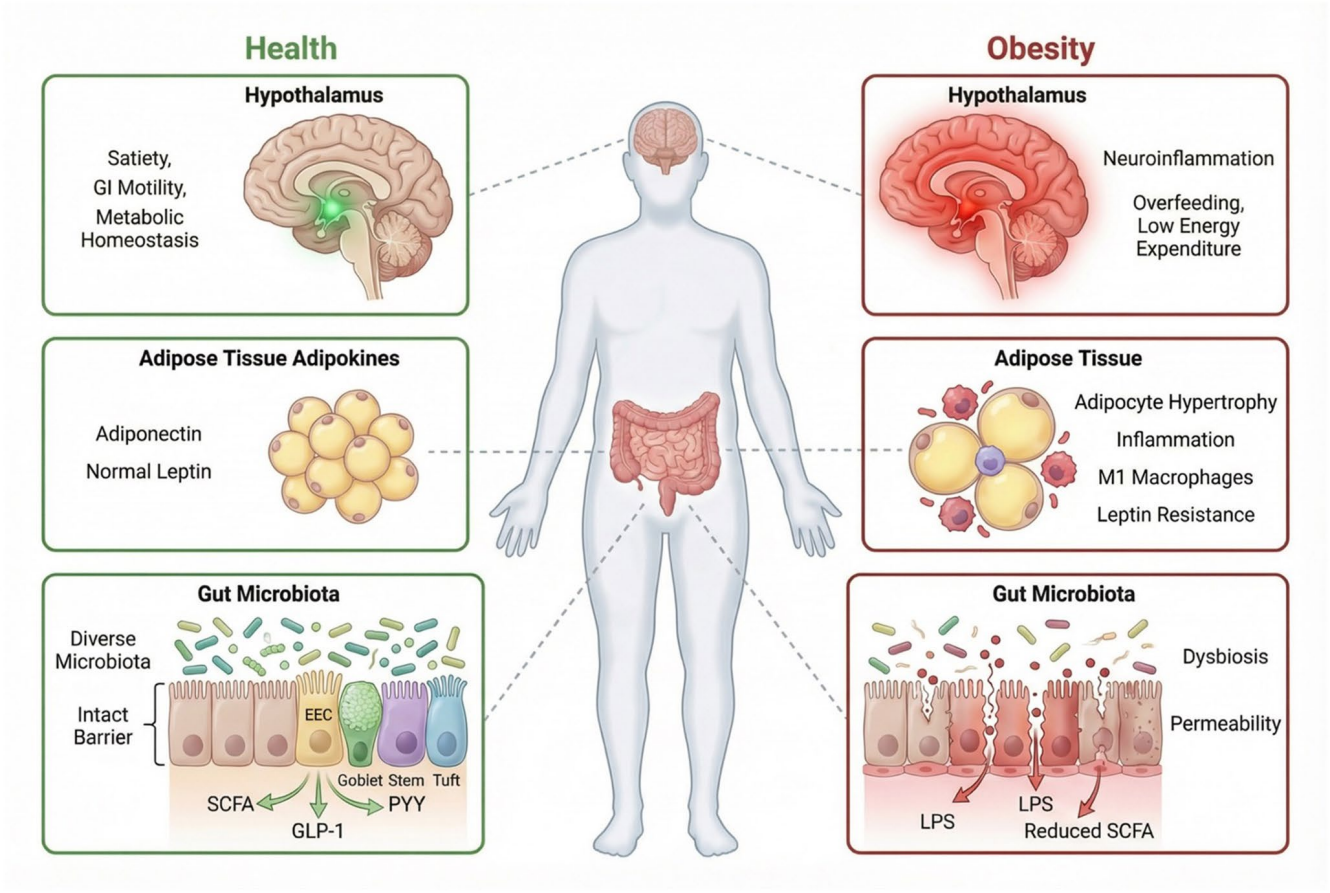
Gut microbiota dysbiosis drives hypothalamic inflammation and metabolic dysfunction in obesity. Schematic comparing healthy (left) and obese (right) states: diverse microbiota and intact gut barrier support hypothalamic leptin sensitivity, SCFA production, and incretin signaling (GLP-1/PYY); dysbiosis induces leaky gut, LPS endotoxemia, reduced SCFAs, hypothalamic neuroinflammation, leptin resistance, and adipose tissue hypertrophy/hyperplasia

Through specialized synapse-like structures called neuropods, EECs form direct contact with vagal afferent fibers, enabling rapid transmission of gut-derived signals to the brainstem [70, 71, 123]. These peripheral cues are further integrated with inputs from brainstem centers, such as the nucleus tractus solitarius (NTS), which receives direct afferent information from the vagus nerve to coordinate appetite and systemic energy balance [124], as shown in Fig. 3. The fact that neuroinflammatory effects induced by LPS-producing bacteria are attenuated by subdiaphragmatic vagotomy supports a vagus-dependent pathway that operates independently of detectable increases in plasma LPS [125]. This multi-modal signaling axis connecting the gut environment to the brainstem and hypothalamus is summarized in Fig. 4.

In response to this chronic metabolic stress, hypothalamic microglia and astrocytes become activated, sustaining the IKKβ and JNK signaling cascades [126]. Experimental evidence indicates that high-fat feeding rapidly induces hypothalamic microinflammation, occurring as early as 24 to 72 h post-exposure and preceding significant weight gain [127]. This early response is characterized by microglial activation and impaired leptin receptor signaling via upregulation of suppressor of cytokine signaling 3 (SOCS3) [127, 128]. These inflammatory pathways impair insulin receptor substrate signaling and reduce leptin receptor-STAT3 activation, contributing to central insulin and leptin resistance. Concurrent activation of endoplasmic reticulum (ER) stress and oxidative stress further compromises neuronal responsiveness [129].

The mechanisms proposed based on rodent models are not currently established in humans, mostly due to methodological limitations in human research. While human studies have reported significant relationships between radiologic markers of hypothalamic inflammation or dysfunction and obesity and visceral adiposity [130–132], we found no direct evidence confirming the link between dysbiosis and hypothalamic inflammation. However, a recent study using histological analysis of *post-mortem* hypothalamic tissue supports a potential association, identifying that hypothalamic inflammation and gliosis were significantly associated with a dietary-fat-induced reduction of *Parasutterella* sp. in the gut [133]. However, these findings are strictly correlational, prospective longitudinal human cohorts are needed to provide mechanistic evidence.

Notably, the brain also exerts peripheral control over energy metabolism through autonomic outputs to white adipose tissue (WAT) [134]. This control is primarily mediated by sympathetic neural pathways arising from centers such as the paraventricular nucleus (PVH), which promote lipolysis via norepinephrine release [135]. Disruption of PVH function can lead to increased lipid accumulation in WAT, suggesting that sympathetic dysfunction favors excessive energy storage and contributes to obesity pathogenesis [136, 137].

In parallel, hypothalamic output also extends to the gastrointestinal tract, where autonomic signaling regulates key determinants of the intestinal environment, including motility, secretion, permeability, and immune tone [138]. Recent experimental evidence demonstrates that chemogenetic activation or inhibition of POMC and AgRP neurons induces rapid, region-specific changes in gut microbiota composition within hours, independently of food intake, in association with increased sympathetic activity and intestinal transcriptional remodeling [138].

At tissue level, sympathetic signaling promotes norepinephrine release in the gut wall, which programs muscularis macrophages via β2-adrenergic signaling and drives local adaptive responses to environmental perturbations [139], providing a mechanistic link between central autonomic output and modulation of the intestinal niche. In humans, this central control is reflected in the requirement of hypothalamic integrity for the full metabolic and microbiota-associated effects of bariatric surgery, as patients with hypothalamic damage exhibit impaired weight loss despite preserved gut hormone responses and show altered microbial adaptations following intervention [140].

Ultimately, the balance between anorexigenic pro-opiomelanocortin (POMC) neurons and orexigenic agouti-related peptide (AgRP) neurons shifts toward a persistent orexigenic tone [141], impairing the brain’s ability to regulate both peripheral metabolism and gut–brain interactions, thereby favoring the maintenance of an obesogenic state [142].

## Adipose tissue immunometabolic remodeling, peripheral dysfunction, and inflammation loop

These central alterations extend to peripheral metabolic tissues, particularly white adipose tissue, where they shape energy storage and inflammatory responses. Under physiological conditions, WAT functions as a dynamic energy reservoir that initially expands through hyperplasia – the recruitment of new adipocytes – in response to energy surplus. However, as the surplus persists, the tissue’s ability to recruit new, functional adipocytes reaches a threshold, and hyperplasia gives way to dysfunctional hypertrophy, a state classically observed in more advanced stages of obesity [143–145]. The regulatory environment of these expansion phases is shaped by cytokines such as TNF-α, which exhibit dual roles: they can stimulate pre-adipocyte proliferation to expand the cellular pool while simultaneously inhibiting terminal differentiation into mature adipocytes [146–148].

As hypertrophy advances, the excessive size of adipocytes restricts blood vessel diameter and impairs local blood flow, leading to tissue hypoxia and triggering extracellular matrix remodeling [149, 150]. This hypoxic microenvironment serves as a critical driver for immunometabolic remodeling; in response to low oxygen states, there is a marked rise in pro-inflammatory adipokines and the polarization of macrophages from an anti-inflammatory (M2) state to a pro-inflammatory (M1) state. These M1 macrophages accumulate within the WAT, often forming crown-like structures around stressed or necrotic adipocytes [151, 152]. The resulting inflammatory environment, dominated by TNF-α, IL-6, IL-8, and MCP-1, directly impairs local and systemic metabolic health, a state further compounded by chronic hyperleptinemia and reduced adiponectin levels that diminish insulin sensitivity and anti-inflammatory signaling [153].

While the cellular mechanics of obesity are well-mapped, the gut microbiota acts as a major modulator of this adipose topography, dictating the storage of lipids in the relatively safe subcutaneous adipose tissue (SAT) or the highly inflammatory visceral adipose tissue (VAT) [154]. As reviewed by Zhong and colleagues (2025), in murine models, SAT accumulation is suppressed by *Limosilactobacillus reuteri*, *Bifidobacterium longum*, and *Bifidobacterium pseudocatenulatum*, which reduce the mRNA expression of *ACC1*, enhance fatty acid oxidation, and promote lithocholic acid (LCA) biosynthesis. Within VAT depots, *Clostridium butyricum*, *Faecalibacterium prausnitzii*, and *Bifidobacterium longum* decrease fat deposition by modulating AMPK, PPARγ, and Wnt10b pathways, inhibiting fatty acid synthase activity and improving insulin resistance. This remodeling is also regulated by the gut microbiota via miR-181, where tryptophan-derived metabolites such as indole tune energy expenditure [155]. Simultaneously, the microbial metabolite desaminotyrosine (DAT) acts as a potent anti-obesity counter-signal, promoting adipocyte-autonomous fat disposal by enhancing sustained lipolysis and increasing fatty acid oxidation through the upregulation of the rate-limiting enzyme CPT1A [156].

At the cellular level, this inflammatory environment directly impairs the insulin signaling cascade. Under physiological conditions, insulin binds to its receptor (IRβ), initiating a cascade through insulin receptor substrates (IRS) and PI3K that leads to the dual phosphorylation of Akt (Thr308 and Ser473) [157]. This process is critical for the translocation of GLUT-4 to the cell membrane for glucose uptake [158]. In obesity, elevated production of TNF-α disrupts this pathway by inhibiting the insulin receptor’s tyrosine kinase activity and promoting inhibitory serine phosphorylation of IRS-1, which reduces GLUT-4 translocation and results in hyperglycemia [159, 160].

Importantly, adipose tissue inflammation does not remain confined to the tissue itself. Cytokines and adipokines enter circulation, influencing distant organs and feeding back to central regulatory circuits. Cytokines TNF-α and IL-1β are innately prone to reinforce their production [161]. This amplifies hypothalamic inflammation and sustains dysregulated autonomic output, establishing a self-reinforcing loop. Once established, this loop stabilizes the obese state and drives the progression of metabolic disease.

The transition from chronic obesity to weight loss involves a complex release of inflammatory mediators. In chronic obesity, the excessive size of hypertrophied adipose tissue restricts local blood flow and traps cytokines and mediators within. While clinical evidence shows that weight loss can reduce macrophage infiltration [162] and normalize adipocyte morphology [163], the improvement in local circulation concurrently allows previously sequestered cytokines to reach the systemic circulation. This acute systemic influx triggers a transient rise in serum inflammatory markers, providing feedback to central circuits that reflects the persistence of the pathological loop even during the early phases of dietary change and weight loss [164, 165].

This phenomenon explains why patients often experience a perceived heightening of systemic inflammation or malaise during the initial stages of a dietary transition. It is especially critical in weight cycling (yo-yo dieting), where repeated phases of dieting and weight regain predispose individuals to accelerate obesity and metabolic comorbidities through phases of systemic inflammation and further harm to gut microbiota health [166]. Moreover, such body mass fluctuations promote an epigenetic memory, that facilitate rebound weight gain regardless of significant improvements in pathophysiology [167], acting as an obesogenic memory phenotype. Consequently, weight cycling is strongly linked with long-term risks for cardiovascular diseases [168, 169], as the repeated release of sequestered inflammatory mediators prevents metabolic resolution and may further prime central regulatory circuits for chronic dysfunction. While current treatments, including GLP-1 and GIP receptor agonists, provide substantial improvements toward obesity and its comorbidities, including dysbiosis, they do not guarantee the maintenance of these benefits once discontinued [170–172].

## Incretin-based modulation of the microbiota-hypothalamus-adipose tissue axis

Persistent inflammatory signaling and metabolic dysregulation has positioned the microbiota–hypothalamus–adipose tissue axis as a key therapeutic target in metabolic disease. In this context, incretin-based therapies have reshaped the treatment strategies, [173]. Recent research indicates that pharmacological GLP-1 and dual GIP/GLP-1 receptor agonists do not simply act as hormone mimetics but have been shown to reshape the intestinal environment, promoting a “lean-associated” microbial phenotype associated with signaling to central and peripheral metabolic centers [174, 175].

A basis of the weight-loss efficacy of GLP-1 analogues is the transition of the gut microbiota into a state that favors specialized metabolite production, such as the microbial metabolite agmatine, which acts as an FXR agonist to inhibit GLP-1 secretion in a feedback loop [176], or the production of citrulline, which mitigates systemic inflammation during sepsis [177].

Semaglutide administration induces profound structural remodeling, specifically enriching acetate-producing bacteria such as *Bacteroides acidifaciens* and *Blautia coccoides* [178, 179]. This taxonomic shift has been associated with changes in hypothalamic energy homeostasis. Elevated levels of gut-derived acetate cross the blood-brain barrier and accumulate in the hypothalamic arcuate nucleus. In the ARC, acetate acts as a signaling ligand for GPR43 located on POMC neurons. Activation of the GPR43/IRS/PI3K/Akt/AMPK/SIRT/FoxO-1/POMC signaling pathway results in the repression of FoxO-1, which in turn enhances the firing of anorexigenic POMC neurons and reduces food intake [178, 180].

Furthermore, semaglutide treatment has been shown to reverse high-fat diet (HFD)-induced hypothalamic neuroinflammation by downregulating the TLR4/MyD88/NF-κB and JNK pathways, restoring central insulin sensitivity [178, 179]. These neuroprotective effects extend to diabetes-associated cognitive decline (DACD), where semaglutide enriches *Alistipes* and *Parabacteroides* to inhibit hippocampal neuron loss [181, 182].

Preclinical studies further demonstrate that semaglutide can remodel gut microbiota structure and that fecal microbiota transplantation (FMT) from treated donors transfers aspects of the metabolic phenotype to recipient animals, supporting a potential role for microbiota in mediating drug effects [180, 183]. In humans, microbiota changes are less consistent at the compositional level. In semaglutide-treated patients, baseline abundance of genera including *Bacteroides*, *Lachnoclostridium*, *Escherichia–Shigella*, and *Prevotellaceae NK3B31 group* is associated with the magnitude of HbA1c reduction, although these associations do not remain significant after multiple testing correction. In contrast, *Alistipes* abundance is associated with reductions in inflammatory markers, including neutrophil counts and neutrophil-to-lymphocyte ratio [184].

In murine models, semaglutide upregulates the expression of genes essential for fatty acid oxidation and thermogenesis in white adipose tissue, including *Ppar-alpha*, *Ppar-gamma*, *Cpt1a*, and *Pgc1-alpha* [180]. These central signals facilitate the browning of subcutaneous fat, effectively increasing systemic energy expenditure. In humans, the SEMALEAN study (2026) confirmed that these changes translate to an 18% reduction in total fat mass and a significant decrease in VAT [185]. Notably, this study observed a decoupling of mass and function. While patients lost absolute lean mass, their handgrip strength improved by 4.5 kg, and the prevalence of sarcopenic obesity fell from 49% to 33%, suggesting that the axis optimizes muscle quality even during rapid weight loss [185].

The stability and efficacy of this axis can be enhanced through adjunctive strategies. The combination of semaglutide with *Akkermansia muciniphila* Akk11 significantly outperforms monotherapy in reducing SAT and VAT [186]. Mechanistically, this synergy is driven by the simultaneous suppression of fatty acid synthesis and the promotion of mitochondrial function across the intestinal, hepatic, and adipose compartments [186]. Furthermore, the introduction of β-glucan-based superabsorbent hydrogels can mimic these effects by slowing gastric emptying and enriching probiotics like *Muribaculaceae*, which correlate with reduced triglyceride synthesis and transport [187]. This is further supported by evidence that engineered probiotics, such as *Clostridium butyricum* modified to produce GLP-1, can restore the gut microbiome to treat hypertension [188].

Dual GIP and GLP-1 agonists like tirzepatide induce broader taxonomic shifts than pure GLP-1RAs, significantly increasing the proportion of beneficial genera such as *Clostridium sensu stricto 1* and *Romboutsia* while suppressing pathogenic taxa like *Erysipelatoclostridium* [189, 190]. The metabolic and renoprotective benefits of tirzepatide are strictly dependent on a functional microbiota. When the gut microbiome is depleted by broad-spectrum antibiotics, the drug’s ability to decrease fasting blood glucose (FBG), reduce food intake, and improve renal function markers, such as serum creatinine and the albumin/creatinine ratio, is markedly attenuated [189]. This confirms the microbiota as an indispensable interface in tirzepatide’s multi-organ protection.

Upon semaglutide withdrawal, a rapid reversal of both microbial and hypothalamic profiles occurs. In a prospective study of 28 women, 71.4% exhibited significant weight regain within 12 weeks of cessation, corresponding to a sharp appetite rebound [191]. This rebound is driven by the reactivation of central orexigenic pathways (AgRP/NPY) and the attenuation of the anorexigenic POMC/MC4R pathway [191]. In parallel, the gut microbiota reverts to a high *Bacillota*-to-*Bacteroidota* ratio, with a significant loss of *Clostridium sensu stricto 1* and a decline in ursodeoxycholic acid levels. This depletion of protective bile acids leads to the downregulation of hypothalamic TGR5 expression, further dismantling the satiety signaling network [191]. Interestingly, while central pathways rebound rapidly, some improvements in hepatic lipid metabolism, which was mediated by maintained AMPK/SIRT1 activation, partially persist, suggesting a temporal lag between central appetite reversal and peripheral metabolic relapse [191].

The microbiota-hypothalamus-adipose tissue axis is modulated by current incretin therapy. By prioritizing acetate-mediated satiety and PPAR-driven lipid oxidation, these drugs achieve remarkable metabolic outcomes. However, the complexity of these paths is emphasized by potential formulation-specific risks, such as chronic SNAC exposure in oral semaglutide depleting primary fermenters like *Muribaculaceae* and *Bacteroidaceae*, leading to reduced butyrate levels and systemic inflammation [192]. Additionally, drug-induced side effects like evodiamine-mediated bone loss linked to the reduction of *Lachnospiraceae* [193] further suggest that future clinical attention must be paid to the secondary effects of microbial remodeling of musculoskeletal health.

## Future perspectives

Although the microbiota-hypothalamus-adipose tissue axis is now recognized as a central regulator of metabolic homeostasis, critical mechanistic gaps remain. Evidence shows that microbial functional outputs outperform taxonomic composition, and future research should focus on causal mapping of microbial metabolites, immune mediators, and neural circuits that determine whether dietary signals are adaptively resolved or consolidated into chronic inflammation. Temporal resolution is particularly needed. Hypothalamic inflammation can precede overt adiposity, yet the relative contributions of postprandial endotoxin dynamics, vagal sensing, cytokine signaling, and endothelial activation remain unclear in humans. Integrative studies combining microbiome functional profiling, metabolic phenotyping, and neuroimaging will be essential to establish causal directionality within this axis.

Interindividual variability further complicates therapeutic translation. Because these functional outputs can arise from distinct microbial configurations, future stratification should integrate microbial functional capacity with host immune sensitivity, adipose distribution, and central insulin/leptin responsiveness. Such axis-based phenotyping may help distinguish immune-dominant from neural-dominant obesity subtypes. Therefore, individualized nutritional strategies aimed at modulating the gut microbiota are essential to mitigate the physiological consequences of dysfunction in this axis. These strategies should focus on restoring microbial metabolic output, particularly pathways involved in SCFA production, which plays a central role in energy homeostasis, inflammatory regulation, and maintenance of intestinal barrier integrity. Future interventions must consider interindividual differences, including genetic, metabolic, age-related, and sex-specific factors. Given the heterogeneity of obesity, generalized therapeutic approaches often yield limited efficacy.

Finally, while incretin-based therapies reshape both microbial ecology and hypothalamic signaling, rapid weight regain after discontinuation suggests incomplete reprogramming of central and peripheral circuits, perhaps reflecting an “obesogenic memory phenotype.” Determining whether durable metabolic remission requires sustained microbial modulation, epigenetic resetting, or combined multi-target approaches represents a key translational priority. Conceptualizing obesity within this integrated signaling axis may guide therapies aimed not only at weight reduction but also at restoring functional signaling across the gut microbiota-brain-adipose tissue network.

## Data Availability

No datasets were generated or analysed during the current study.
